# Editorial: Antibacterial effects and mode of action of new active substances against drug resistant pathogenic bacteria

**DOI:** 10.3389/fchem.2024.1526451

**Published:** 2024-11-22

**Authors:** Abderrahmen Merghni

**Affiliations:** Laboratory of Antimicrobial Resistance LR99ES09, Faculty of Medicine of Tunis, University of Tunis El Manar, Tunis, Tunisia

**Keywords:** active substances, chemical characterization, antibacterial effects, mode of action, biofilm

Antimicrobial resistance (AMR) is still one of the most difficult concerns threatening human’s health across the world and represents one of the top ten worldwide public health challenges confronting humanity according to World Health Organization. Consequently, the economic burden of AMR is substantial. In fact, long-term disease not only increases the risk of mortality and incapacity but also lengthens hospital stays, necessitates the use of more expensive medications, and puts a strain on the finances of those affected.

Without efficient antimicrobials, infections would be more difficult to treat in modern medicine, notably for immune-compromised patients. Numerous lines of research are developed into new classes of molecules aimed at new “targets” of action in bacteria, in order to circumvent bacterial resistance mechanisms.

This Research Topic offers a platform to highlight the effectiveness of new active substances in pathogenic bacteria and particularly their anti-biofilm potential. It aim to investigate the effect of new anti-infection agents from natural (such as plant extracts, probiotics, etc.) or chemical resources, against drug resistant pathogenic bacteria, as-well as exploration of their different mode of actions and competitive effects.

The paper of Heena et al., investigated the antibacterial potential of Quinic acid (QA) and its derivatives isolated from the ethyl acetate fraction of a methanol extract of *Citrus reticulata* dropped fruits, They are subjected to molecular docking studies to predict their binding affinity with DNA gyrase, transpeptidase, and β-lactamase; their synergistic interaction with standard antibiotics; the antibiofilm activity of the most effective treatment; and an analysis of their toxicity and drug-like properties using absorption, distribution, metabolism, excretion, and toxicity (ADMET) analysis. Results from this paper revealed that 3,4-o-isopropylidenequinic acid-1,5-lactone (QA1) showed maximum antibacterial potential through damage to the bacterial cell membrane along with inhibition of biofilm formation and showed synergistic interaction with streptomycin. The compound belongs to class 6 toxicity and is safe to use. The compound also has suitable physiochemical properties and pharmacokinetic profile and fulfills all the drug likeliness parameters. Hence, it can be categorized as a potential antibacterial drug candidate.

The antibiotic resistance exhibited by bacterial biofilms, along with their role in the development of infectious diseases in humans, underscores the necessity for the creation of innovative and effective antibacterial and anti-biofilm agents. The study conducted by Aithani et al., designed and synthesized a novel class of antibacterial agents, consisting of 1,4-benzothiazine-based bisamide derivatives, with the aim of targeting bacterial peptide deformylase (PDF) to effectively address *S. aureus* infections. Through computational design, a novel identified series of bisamide derivatives of (1,4)-benzothiazine-3-one exhibit antibacterial and antibiofilm properties against *Staphylococcus aureus*. Molecular modeling studies targeting the peptide deformylase of *S. aureus* revealed significant interactions of the compound that contribute to its efficacy. Additionally, the effectiveness of these compounds in inhibiting biofilm formation on urinary catheters has been demonstrated.

As part of the same plan to combat bacterial biofilm, the study of investigated Singh et al., the potential of cell free preparations of lactobacilli isolated from breast milk (HM; n = 11) and infant faecal (IF; n = 15) samples to impact the growth of *S. aureus* and *Pseudomonas aeruginosa* biofilms. The results of this study support the hypothesis that the therapeutic efficacy of lactobacilli against food-borne and clinical pathogens primarily arises from the disruption of interfacial interactions, including cell-to-cell and cell-to-surface interactions, or from the secretion of exo-metabolites that compromise the structure and organization of biofilms. Furthermore, the various strains of lactic acid bacteria exhibited unique capabilities to hinder biofilm formation by inhibiting the initial adhesion process.

The study of Mahdizade Ari et al., assessed the microbiological, immunological, periodontal, and clinical outcomes after examining the impact of photodynamic therapy on individuals with chronic periodontitis. Photodynamic therapy has been a popular treatment for periodontal disorders due to its strong results, as demonstrated by *in vitro* and *in vivo* research. Compared to scaling and root planing, photodynamic therapy is considered a promising new antibacterial technique, adjunctive, and low-cost therapeutic method that is effective in tissue repair, reducing chronic periodontitis, reducing inflammation, and well-tolerated by patients.

This compilation is of utmost significance owing to its diverse contributions to our scientific understanding and practical implementations. It provides a better understanding of the specificity of each of the antimicrobial substances studied, broadening our understanding of their modes of action in bacterial cells and their effectiveness against the particular resistant structure that is the biofilm. Ultimately, these studies contribute to our broader scientific goals, helping us to increase our knowledge of new and effective antibacterial and anti-biofilm agents that can be potentially valorized.

